# Independent and joint contributions of inadequate antenatal care timing, contacts and content to adverse pregnancy outcomes

**DOI:** 10.1080/07853890.2023.2197294

**Published:** 2023-04-24

**Authors:** Mahama Saaka, Issahaku Sulley

**Affiliations:** School of Allied Health Sciences, University for Development Studies, Tamale, Ghana

**Keywords:** Inadequacy of antenatal care utilization, preterm delivery, low birth weight, Northern Ghana

## Abstract

**Background:**

Poor quality and inadequate of antenatal care (ANC) visits during pregnancy may increase the risk of preventable adverse pregnancy outcomes. We tested the hypothesis that the adequacy of ANC utilization combined with quality of ANC services will reduce the risk of low birth weight (LBW) and preterm delivery (P T D) in the Tamale metropolis of Ghana.

**Materials and methods:**

A facility-based analytical cross-sectional study was conducted on a sample of 553 postpartum women who had delivered within the last 12 months prior to the study. The overall utilization of ANC services was measured in terms of ANC timing, contacts, and content (TCC) of essential ANC services. The sample was drawn using systematic random sampling procedure. Primary data was collected from mothers by administering a structuredquestionnaire while the secondary data was extracted from individual records.

**Results:**

After controlling for confounders, women who had adhered to all WHO recommendations in terms of ANC timing, frequency and content were 71 % protected from PTD, AOR = 0.29 (95 % CI: 0.15, 0.59) and 56 % protection from LBW AOR = 0.44 (95 % CI: 0.23, 0.83).

**Conclusion:**

Individually and jointly, inadequate ANC contacts and content associatedsignificantly with preterm delivery than LBW.

**Key messages**
- Limited evidence exists on the joint effect of ANC services timing, contacts and content on adverse pregnancy outcomes.
- Total adherence to recommended ANC initiation, attendance and receipt of essential services had greater protection against PTD and LBW, compared to any single element/component of ANC
- Women who had adequate overall ANC services utilization in terms of timing, contacts and content were 71 % protected from PTD, AOR = 0.29 (95 % CI: 0.15, 0.59) and 56 % protection from LBW AOR = 0.44 (95 % CI: 0.23, 0.83).

## Introduction

Low birth weight (LBW), and preterm delivery (PTD) are of public health significance because they incur high costs to health care services and also contribute to neonatal morbidity and mortality [1–3]. PTD (<37 weeks of gestation) exposes infants to greater risk of postnatal long-term cognitive, behavioral, social, emotional, and neurodevelopmental difficulties [4]. Similarly, birth weight greatly determines a child’s chances of survival, and healthy postnatal growth [5]. LBW (<2500 g) contributes 60 to 80% of all neonatal deaths [6] while PTD contributes to ∼80% of perinatal mortality [7]. To achieve the targeted Sustainable Development Goals 3.1 and 3.2, any measure that will reduce the prevalence of adverse birth outcomes such as LBW and PTD should receive priority attention.

The persistent high prevalence of adverse pregnancy outcomes in many settings is an indication that not all risk factors have been identified and addressed. As a result, many health care systems continue to seek for effective measures to manage this situation. One of such measures is the promotion of antenatal care (ANC) which serves as a platform for the delivery of health and nutrition interventions during pregnancy. Timely initiation and frequent ANC attendance as recommended by the World Health Organization (WHO) are some of the major strategies available to improve pregnancy outcomes. This is because, timely initiation and frequent ANC visits give health service providers an opportunity to deliver vital services including treatment of pregnancy-induced hypertension, tetanus immunization [8–10], prophylaxis and micronutrient supplementation [11,12]. All of these are preventative measures that have the potential to improve pregnancy and neonatal outcomes [13].

Previously, Ghana adopted the WHO Focussed ANC (FANC) Framework, which recommended expectant mothers to have at least four ANC visits throughout pregnancy [14]. This policy was taken to help reduce high maternal mortality and to improve access and quality of ANC services. Routine ANC services are organized at different levels including health centres, community clinics, private and public hospitals, or polyclinics. ANC services are provided by trained midwives, doctors, and nurses in health facilities.

However, in 2016, the WHO revised the ANC guidelines to recommend at least eight ANC contacts instead of four, with the first contact to be made within the first 12 weeks of gestation [15–17]. By the revised WHO ANC recommendations, two of the eight contacts should be in the second trimester and five in the third trimester [18].

In Ghana 85% of pregnant women attend at least one antenatal visit with a skilled provider and only 55% of pregnant women in the rural areas are able to attend ANC at least four times during pregnancy [8,19]. In Northern Ghana, where this study was conducted, late initiation of ANC services (>12 weeks gestation) is reported to be widespread [20]. Poor quality and inadequate utilization of ANC services during pregnancy may increase the risk of preventable adverse pregnancy outcomes but how these collectively influence these outcomes remain understudied. Though some studies in Ghana including [21–23] have assessed the individual effect of ANC components (that is, timing, frequency and content) on pregnancy outcomes, none but one [24] has investigated the joint effect of these variables. More especially, little is documented regarding the protective effect of adequate utilization of the recommended eight or more ANC contacts on adverse pregnancy outcomes. It is in the light of the foregoing; we tested the hypothesis that the adequate timing, contacts combined with quality of ANC services will reduce the risk of LBW and PTD in the Tamale metropolis of Ghana.

## Materials and methods

The methods section is written based on the Strengthening the Reporting of Observational Studies in Epidemiology (STROBE) statement [25].

### Study setting

The study was carried out in the Tamale Metropolis of the Northern Region of Ghana. The metropolis has 47 health facilities including the Tamale Teaching Hospital that deliver health care to the people of the metropolis and beyond.

### Study design, population and sampling

We conducted a facility-based analytical cross-sectional study on a population comprising all postpartum women who had attended ANC and delivered within the past 12 months in a health facility preceding the study. A list of health facilities in the Metropolis was compiled from which 10 of them were carefully chosen by means of simple random sampling. In each selected health facility, the study sample was extracted using systematic random sampling procedure. Names of the mother-child pairs in the postnatal care (PNC) register were used as sampling frame.

Using the formula for estimating single population proportion, the minimum possible sample size of 485 was estimated at 95% confidence level, a margin of error (α = 0.05), a population proportion of LBW to be 30% [26], and a design effect of 1.5 . Making room for 15% contingency to cater for unexpected events including damaged and incomplete questionnaire, the sample size was adjusted to 558.

### Data collection

Both primary and secondary data were collected. A structured and pre-tested questionnaire designed by the investigators and based on previous literature [27–30], was used to collect the primary data from postpartum mothers. The secondary data which were mainly obstetric data were collected by reviewing the antenatal and postnatal health records of the mother. The obstetric data included parity, gravidity, timing of first ANC visit, content, and frequency of utilizing ANC services, gestation at delivery and mode of delivery. Other maternal information obtained were socio-demographic characteristics (e.g. age, occupation, education, household wealth index), weight and height of mother, maternal behaviours and health status during last pregnancy (e.g. malarial infection, smoking, alcohol consumption), haemoglobin, systolic and diastolic blood pressure.

### Measurement study variables

The main outcome measures were LBW and PTD and the principal independent variable was ANC utilization which was measured using a modified version of the adequacy of prenatal care utilization (APNCU) index [31]. The APNCU index gives a precise and comprehensive measurement of ANC [32]. The WHO recommends antenatal visits of at least eight times during pregnancy and to initiate ANC in the first trimester [17]. In this study, the overall utilization of ANC services was measured in terms of timing of ANC, frequency of ANC visits and ANC content received in accordance with 2016 revised ANC WHO guidelines. Thus, to be considered having adequate ANC, a pregnant woman must have initiated ANC in the first trimester, made at least 8 contacts and received 10 ANC recommended services.

The other independent variables of interest were the frequency of ANC visits and the content of ANC services received. Frequency of visits were classified as less than eight visits and at least eight visits as per the WHO’s revised recommendations for an uncomplicated pregnancy [17].

The content of ANC services received which serves as a proxy measure of ANC quality received was measured by the extent to which each pregnant woman received essential ANC services [14,33]. The set of 10 recommended health and nutrition services the mothers received during pregnancy were checking of mother’s weight at least two times, the height of mother measured on first ANC visit, blood pressure taken at least three times, urine and blood samples examination performed at least once, receipt of malaria prophylaxis at least five doses, health and nutrition education sessions at least four times on possible danger signs/complications of pregnancy, TT injection at least once, monthly iron/folic acid supplementation, and monthly measurement of fundal height/ultrasound, foetal heart beat, and foetal movement count.

The survey tool used in assessing ANC quality in this study is universally accepted and has been widely used in the literature [17,27–29,33]. ANC content was calculated as a composite index based on responses to 10 questions about whether the pregnant women received the 10 recommended ANC services during their last pregnancy. Each positive response had a score of 1 and negative response = 0. The details of this index which ranged from 0 to 10 have been previously described [34,35]. ANC content was categorized as low if any of the 10 recommended services was not received by the pregnant woman (coded ‘0′); and high ANC content was assigned to respondents who received all the 10 recommended services (coded ‘1′).

### Data analysis

The data set was cleaned and coded for analysis using Statistical Package for Social Science (SPSS) version 22 (SPSS Inc, Chicago). Univariate and multivariable logistic regression analysis was used to assess the associations between the exposure predictor variables and adverse pregnancy outcomes while controlling for potential confounders. Adjusted odds ratio (AOR) with a 95% Confidence Interval (CI) was computed to identify statistically significant predictors of adverse birth outcomes. Confounding variables adjusted for included household socioeconomic status, infant’s sex, place of residence, marital status, maternal age and education, parity, and gravidity.

### Ethics declarations

The Institutional Review Board (IRB) of the Navrongo Health Research Centre provided ethical clearance for the study (Reference No. NHRCIRB372). The Tamale Metropolitan Health Directorate granted approval to use the Sub-Metropolitan health facilities as the study sites for data collection. Informed consent was obtained from the study participants prior to data collection.

## Results

### Socio-demographic characteristics of the respondents

The socio-demographic characteristics of the respondents in the study sample are shown in Table 1. The mean maternal age was 29.43 ± 6.26 years. While most participants representing 95.8% were married, 49.9% of them never had any formal education. In terms of household wealth index, 74.7% were classified as high and 78.5% were non-salary workers.

**Table 1.** Socio-demographic characteristics of the respondents (*N* = 553).

Characteristic	Frequency (n)	Percentage
Age of mother (years)		
<25 years	126	22.8
25–34 years	303	54.8
≥35 years	124	22.4
Marital status		
Not married	23	4.2
Married	530	95.8
Ethnicity		
Dagomba	409	74.0
Mamprusi	55	9.9
Gonja	48	8.7
Akan	19	3.4
Others	22	4.0
Religion		
Muslim	465	84.1
Christian	88	15.9
Maternal education		
No formal education	276	49.9
Low (primary & junior high school)	152	27.5
High (at least senior high school)	125	22.6
Maternal occupation		
Salary worker	64	11.6
Non- salary worker	434	78.5
Unemployed	55	9.9
Type of residence		
Urban	199	36.0
Rural	183	33.1
Peri-urban	171	30.9
Household wealth index		
Low	140	25.3
High	413	74.7

### Utilization of ANC services and birth outcomes

The uptake of adequate ANC services among pregnant women was low. The proportion of women who made at least eight ANC contacts during pregnancy and making the first visit in the first trimester (that is, early utilization of ANC services) of pregnancy was 27.1% (95% CI: 23.5–31.1). The proportion of women who initiated ANC in the first trimester of pregnancy was 71.1%. The proportion of pregnant women who adhered to WHO recommended ANC initiation, attendance and receipt of essential services was only 24.1% (95% CI: 20.4–27.7). The overall prevalence of LBW and PTD were 93 (18.3%) and 102 (18.4%) respectively. The prevalence of post-term delivery was 8.1% (Table 2).

**Table 2.** Utilization of antenatal care services and birth outcomes.

Indicator	Frequency (n)	Percentage (%)	Mean ± SD
Gestational age at first ANC visit (weeks)			
Mean			12.8 ± 4.3
First trimester (<13)	393	71.1	
Second trimester (13–16)	55	9.9	
Third trimester (>16)	105	19.0	
Beyond first trimester (>12)	160	28.9	
Frequency of ANC contacts			
<8	400	72.3	
≥8	153	27.7	
Adhered to timely initiation and contacts			
Inadequate	403	72.9	
Adequate	150	27.1	
ANC content			
Low (less than 10 recommended services)	221	40.0	
High (receiving all 10 recommended ANC services)	332	60.0	
Content and timing of care in pregnancy (CTP)			
Inadequate	235	42.5	
Adequate	318	57.5	
Adherence to timing, contacts and content (ANC Adequacy)			
No	420	75.9	
Yes	133	24.1	
Birth weight (grams)			
Mean			2986.6 ± 588
LBW (<2500)	93	18.3	
≥2500	414	81.7	
Very low birth weight (VLBW) (<1500 g)	0	0.0	
Gestation at delivery (weeks)			
Mean			37.9 ± 1.8
Post-term delivery (>41 weeks)	45	8.1	
Full term (37–41) weeks	406	73.4	
Preterm delivery prevalence (<37)	102	18.4	
Late preterm delivery prevalence (34.1–36.9)	98	17.7	
Moderate preterm delivery prevalence (32–34)	4	0.7	
Very preterm delivery prevalence (28–32)	0	0.0	
Extremely preterm delivery prevalence (<28 weeks)	0	0.0	

### Prevalence of adverse pregnancy outcomes stratified by uptake of ANC services

Utilization of ANC services indicators associated with PTD than LBW (Table 3). Women who initiated ANC in the first trimester had less PTD compared to women who started ANC after the first trimester and there was also a positive association between timely initiation of ANC and LBW. This means as adequacy of ANC decreases, there was an increase in the risk of preterm birth. The receipt of high ANC content also associated negatively with PTD. Frequency of ANC attendance at least 4 visits based on previous WHO ANC guidelines significantly associated with PTD and LBW. However, the association between at least 8 contacts and LBW was weak in this study sample.

**Table 3.** Prevalence of adverse delivery outcomes stratified by uptake of ANC services.

Characteristic	Adverse pregnancy outcome			
	Preterm n (%)	Test statistic	LBW n (%)	Test statistic
Timing of ANC visits				
Beyond first trimester	45 (28.1)	*χ^2^* = 14.0 , *p* < .001	34 (24.5)	*χ^2^* = 4.8 , *p* = .03
First trimester	57 (14.5)		59 (16.0)	
Frequency of ANC				
<4	47 (29.4)	*χ^2^* = 17. 9, *p* < . 001	35 (26.1)	*χ^2^* = 7. 4, *p* = .007
≥4	55 (14.0)		58 (15.5)	
Frequency of ANC				
<8	89 (22.3)	*χ^2^* = 13. 9, *p* < . 001	73 (20.5)	*χ^2^* = 3. 7, *p* = . 05
≥8	13 (8.5)		20 (13.2)	
ANC content				
Low (received less than 10 recommended services)	57 (25.8)	*χ^2^* = 13. 2, *p* < . 001	42 (22.8)	*χ^2^* = 3.9, *p* = . 05
High (received all 10 recommended ANC services)	45 (13.6)		51 (15.8)	
Adherence to ANC services recommendations				
None (late timing, < 8 contacts and poor content)	44 (30.6)	*χ^2^* = 22. 1, *p* < . 001	33 (26.4)	*χ^2^* = 8.1, *p* = . 04
Timing only	14 (19.2)		8 (14.5)	
Timing + frequency	31 (15.3)		34 (17.5)	
Timing + frequency + content	13 (9.8)		18 (13.5)	

### Predictors of preterm birth and LBW

Several predictors including age of mother, mother’s educational level, birth interval, timing of first ANC visit, frequency of ANC attendance, adequacy of ANC attendance, gravidity, parity, occupation of mother, maternal age, timing of first ANC attendance, and household wealth index were modelled to determine which factors predicted PTD and LBW. Multiple logistic regression analysis showed that the consistent significant predictors of PTD were ANC services utilization, parity and type of employment (Table 4).

**Table 4.** Logistic regressions predicting odds of preterm birth and low birth weight.

Variable	Preterm birth		Low birth weight	
	COR (95% CI)	AOR (95% CI)	COR (95% CI)	AOR (95% CI)
Adherence to ANC services recommendations				
Timing only	0.54 (0.27, 1.07)	0.71 (0.35, 1.45)	0.48 (0.20, 1.11)	0.46 (0.19, 1.09)
Timing + frequency	0.41 (0.24, 0.69)**	0.47 (0.27, 0.80)**	0.59 (0.34, 1.02)	0.58 (0.33, 1.02)
Timing + frequency + content	0.25 (0.13, 0.48)***	0.29 (0.15, 0.59)***	0.44 (0.23, 0.83)**	0.44 (0.23, 0.83)**
None (late timing, < 8 contacts and poor content)	Reference	Reference	Reference	Reference
Maternal height				
140–154.99 cm	1.34 (0.75–2.41)	1.09 (0.59, 2.02)	1.96 (1.10, 3.51)*	1.83 (0.99, 3.35)
155–159.99 cm	1.13 (0.69, 1.85)	1.08 (0.64, 1.81)	1.03 (0.61, 1.75	0.93 (0.53, 1.11)
> =160cm	Reference	Reference	Reference	Reference
Maternal occupation				
Government worker	0.41 (0.18, 0.93)*	0.72 (0.30, 1.73)	0.30 (0.11, 0.86)*	0.40 (0.13, 1.20)
Non-government worker	0.30 (0.17, 0.55)***	0.44 (0.23, 0.85)*	0.68 (0.35, 1.34)	0.70 (0.35, 1.43
Unemployed	Reference	Reference	Reference	Reference
Classification of gravidity				
Primigravida	3.17 (1.91, 5.27)***	1.44 (0.22, 9.32)	1.33 (0.77, 2.27)	0.46 (0.09, 2.50)
Secundigravida	1.84 (1.05, 3.21)*	2.41 (0.42, 13.67)	1.10 (0.63, 1.95)	1.08 (0.27, 4.36)
Multigravida	Reference	Reference	Reference	Reference
Classification of parity				
Primiparous	3.23 (1.95, 5.36)***	2.77 (1.63, 4.68)***	1.51 (0.89, 2.55)	1.77 (0.33, 9.54)
Secundiparous	1.62 (0.92, 2.85	1.65 (0.93, 2.94)	1.07 (0.60, 1.89)	0.95 (0.24, 3.84)
Multiparous	Reference	Reference	Reference	Reference
Type of residence				
Urban	0.93 (0.54, 1.61)	1.05 (0.59, 1.87)	1.54 (0.85, 2.80)	1.56 (0.85, 2.87)
Rural	1.27 (0.75, 2.16)	1.28 (0.72, 2.26)	2.47 (1.36, 4.46)**	2.49 (1.36, 4.56)**
Peri-urban	Reference	Reference	Reference	Reference
Marital status				
Unmarried	2.0 (0.80, 5.0)	1.73 (0.64, 4.68)	3.31 (1.37, 7.98)**	3.06 (1.22, 7.68)**
Married	Reference	Reference	Reference	Reference
Age of mother (years)				
Under 25	3.26 (1.71, 6.20)***	1.62 (0.64, 4.09)	1.36 (0.72, 2.58)	1.30 (0.50, 3.43)
25–34	1.18 (0.64, 2.17	1.05 (0.53, 2.09	0.83 (0.47, 1.47)	0.86 (0.45, 1.63
35^+^	Reference	Reference	Reference	Reference

*Notes:* AOR (95% CI): Adjusted odds ratio at 95% confidence level. C OR (95% CI): Crude/unadjusted odds ratio at 95% confidence level.

*p < .05; **p < .01; ***p < .001.

After controlling for confounders, women who had adhered to all recommendations in terms of ANC timing, frequency and content were 71% protected from PTD, AOR = 0.29 (95% CI: 0.15, 0.59). Primiparous women were 2.8 times more likely of delivering preterm AOR = 2.77 (95% CI: 1.63, 4.68), compared to multiparous women. Non-government workers were 56% protected from delivery of preterm babies AOR = 0.44 (95% CI: 0.23, 0.85), compared to their counterparts who were unemployed.

The analysis showed that inadequacy of ANC, rural residence and unmarried women were consistent predictors of LBW. After adjusting for potential confounding variables, adequate ANC utilization was retained in the final model and provided 56% protection from LBW AOR = 0.44 (95% CI: 0.23, 0.83). Comparatively, women resident in rural areas were 2.49 times AOR = 2.49 (95% CI 1.36, 4.56) more likely to be at risk of delivering LBW babies than those in peri urban. Compared to married women, unmarried women were 3.1 times more AOR = 3.06 (95% 1.22, 7.68) likely of delivering LBW babies.

### Factors associated with receiving adequate ANC services

The results indicated several socio-demographic characteristics including religion, maternal age at registration, education, occupation, and wealth index significantly associated with adequacy of ANC utilization (Table 5).

**Table 5.** Relationship between receiving adequate ANC services and selected factors.

		ANC services utilization		
	N	Inadequate	Adequate	
Variable		n (%)	n (%)	Test statistic
Age of mother (years)				
Under 25	126	66 (52.4)	60 (47.6)	Chi-squared (χ^2^) = 20.5, *p* < .001
25–34	303	93 (30.7)	210 (69.3)	
35^+^	124	37 (29.8)	87 (70.2)	
Maternal education				
No formal education	276	112 (40.6)	164 (59.4)	*χ^2^* = 12.6, *p* = .002
Low (primary & JHS)	152	56 (36.8)	96 (63.2)	
High (at least SHS)	125	28 (22.4)	97 (77.6)	
Place of delivery				
Home	48	34 (70.8)	14 (29.2)	*χ^2^* = 28. 8, *p* < .001
Health facility	505	162 (32.1)	343 (67.9)	
Religion				
Islam	465	177 (38.1)	288 (61.9)	*χ^2^* = 8.8, *p* = .003
Christianity	88	19 (21.6)	69 (78.4)	
Mother’s occupation classification				
Salary worker	64	6 (9.4)	58 (90.6)	*χ^2^* = 26.7, *p* < .001
Non-salary worker	434	161 (37.1)	273 (62.9)	
Unemployed	55	29 (52.7)	26 (47.3)	
Gravidity				
Primigravida	130	67 (51.5)	63 (48.5)	*χ^2^* = 19.6, *p* < .001
Secundigravida	122	40 (32.8)	82 (67.2)	
Multigravida	301	89 (29.6)	212 (70.4)	
Classification of parity				
Primiparous	138	67 (48.6)	71 (51.4)	*χ^2^* = 14.4, *p* = .001
Secundiparous	130	44 (33.8)	86 (66.2)	
Multiparous	285	85 (29.8)	200 (70.2)	
Classification of wealth index				
Low	140	73 (52.1)	67 (47.9)	*χ^2^* = 22.8, *p* < .001
High	413	123 (29.8)	290 (70.2)	
Time to walk to the nearest health facility for treatment				
Less than 30 min	251	47 (18.7)	204 (81.3)	*χ^2^* = 56.1, *p* < .001
At least 30 min	302	149 (49.3)	153 (50.7)	

Adequate utilization of ANC services was more associated with pregnant women who were Christians, compared to Muslim women. Women who had attained at least second cycle education were far more likely (77.6%) to achieve adequate ANC services utilization, compared with women who had no formal education. For occupation, a greater proportion of salary workers utilized ANC services adequately than women that were unemployed. Similarly, women of high household wealth index were more likely to have adequate ANC services utilization than those with lower wealth index. Older women as well as those of high gravidity and parity were more likely to attain adequacy of the services than those who were younger.

## Discussion

In this study, key components of ANC services utilization and their association with preterm birth and LBW were compared. The results showed that individually and jointly, inadequate ANC timing, frequency and content were significantly more associated with PTD than LBW.

### Prevalence of adverse pregnancy outcomes

The prevalence of LBW was 93 (18.3%). The prevalence of LBW as reported by the 2014 Ghana Demographic and Health Survey was 10% [36] while UNICEF reported the prevalence to be 13.0% [37]. However, a similar study carried out in the same study location of Northern Ghana showed that the prevalence of LBW was 29.6% which is relatively higher than what was obtained in this study. A possible explanation for this discrepancy may be due to the fact that study include participants from the Sagnarigu District and Savelugu-Nanton Districts which are mainly peri-urban whilst the present study was limited to the Tamale Metropolis. Pregnant women resident in the rural areas may have limited access to adequate counselling and personnel compared to their counterparts in well-endowed urban settings where ANC services would be better might account for the observed lower LBW.

The prevalence of PTD in the study was quite high. PTD is an important risk factor for under-five morbidity and mortality [38]. In our study sample, 18.4% of deliveries were preterm which is consistent with the 18.9% reported in a recent study from the Korle-Bu Teaching Hospital (KBTH) in southern Ghana [39]. Globally, preterm birth is reported to be between 5 and 18% of all births, most of which occur in the developing countries [40].

### Effect of ANC timing, frequency and content on pregnancy outcomes

The results of this study showed that, total adherence to the recommended ANC timey initiation, frequent attendance, and receipt of essential services (adequate ANC utilization) had greater protection against PTD and LBW, compared to any single element/component of ANC. Our finding is congruent with a recent study which reported that women who reported high quantity and high quality of ANC had lower odds of giving birth to a LBW child in India, Nepal and Pakistan, compared to women reporting both low quantity and low-quality ANC [41]. The finding also confirms other earlier studies conducted elsewhere which found that pregnant women need not only timely and frequent ANC but also care that covers basic processes of care to protect against adverse pregnancy outcomes including PTD and LBW [28,42–50].

Evidence from previous studies suggests that late initiation of ANC care has been found to be associated with LBW, premature labour, preterm babies and intra-uterine deaths [51,52]. The risk of preterm birth is reported to be higher in women who initiate ANC beyond 12 weeks of gestation in comparison to women who started care in the first trimester of pregnancy [22,53,54].

One possible explanation for these findings is that adequate utilization of ANC services makes it possible for health professionals to take preventive measures against adverse pregnancy outcomes. For example, check-ups are made which can help to identify urgent at-risk pregnancies including intrauterine growth restriction for a timely intervention. Nutrition education as well as supplementation of nutrient fortified foods are usually provided at ANC. Furthermore, uptake of ANC services afford the opportunity for early detection and treatment of diseases such as syphilis, malaria, HIV/AIDS, high blood pressure, gestational anaemia and intestinal helminthiasis infection that could affect foetal growth.

During ANC, pregnant women also have access to information on danger signs of pregnancy, birth preparedness and complication readiness. Pregnant women may therefore seek early treatment for any potential pregnancy related problems. This suggest therefore that adequacy of ANC can help protect against adverse pregnancy outcomes through preventative measures as well as early management of complications. In summary, early detection and treatment of pregnancy complications is reported to be associated with reduced perinatal mortality, small for gestational age (SGA) and LBW newborn [43,55,56].

There is however, a continued debate on the association between ANC and pregnancy outcomes as some studies including randomized trials and meta-analysis have found no relationship between ANC services utilization and adverse pregnancy outcomes such as preterm birth [57–60]. The lack of positive association between ANC and birth weight reported in some earlier studies may have been due to the non-inclusion of the timing, frequency as well as the content in quantifying the utilization of ANC services. The effect of inadequate ANC on PTD and LBW may vary depending on how the utilization of ANC services is quantified [61].

In our study sample, women who adhered to recommended ANC timing and attendance frequency were less likely to deliver preterm, compared to their counterparts who failed to meet these recommendations. The association was even greater for women who received essential ANC services in addition to early ANC timing and frequency of attendance. This is consistent with some studies which reported that women who received adequate level of care content were protected from PTD and BW [23,54,62].

In this study, ANC quality was measured in terms of adequacy of content of services received during ANC contacts. Quality of ANC has been measured variously and most of the studies defined ANC quality as receipt of all essential components of ANC services such as blood pressure measurement, blood test, urine test, informed on possible complications, counselling on nutrition, and advice on birth preparedness plan during pregnancy [34,35,63]. The need to incorporate ‘respectful maternity care’ in assessing ANC quality is however gaining grounds to establish what is known as person-centred ANC (PCANC). According to WHO framework, the PCANC dimension of ANC quality measures experience relating to effective communication, respect, dignity, and emotional support [64].

### Predictors of adverse pregnancy outcomes

Inadequate ANC services utilization, being unmarried and rural residency were the key predictors of LBW while inadequate uptake of ANC, mother’s occupation and primiparity consistently predicted PTD.

High prevalence of LBW was observed in rural areas and this finding is consistent with findings of similar studies which have also identified rural residential status to be significantly associated with increased risk of LBW [26,65]. The rural-urban differences in birth weight may be due to several factors including poor access to health services [65–67] in rural areas and socio-economic differentials [68–70]. However, some earlier studies in the Brong Ahafo Region of Ghana, Ethiopia and Bangladesh reported an increased risk of LBW rather among women from urban settings [71–73].

Women who could not adhere to all three recommended components of ANC (timely initiation, at least eight contacts and receipt of essential services) had greater odds of delivering preterm babies. Our finding is in agreement with previously reported associations in Ghana and other low- and middle-income countries including Uganda, Kenya and Bangladesh [45,74–78].

Primiparous women had greater odds of delivering preterm, compared to multiparous women. This finding corroborates that reported in some countries including Denmark where the risk of preterm deliveries in primiparous women was higher compared with multiparous women [79]. It has also been reported that, compared with secundiparous women, nulliparous women have greater risk of preterm birth [80].

In the bivariate analysis, we observed that women younger than age 25 years were more likely to have a PTD, compared to women older than age 25. A large study in n Denmark showed that women aged 20 to 29 years have increased risk of PTD, compared with women aged at least 35 years [80]. Several other studies have shown strong associations between advanced maternal age and PTD [39,81–83] and so younger women of the reproductive age should be targeted to reduce PTD rates.

Working pregnant women were more protected from delivery of preterm babies, compared to their counterparts who were unemployed. In other words, unemployed pregnant women were more likely to deliver preterm. There appears to be much controversy in the literature regarding the influence of unemployment on pregnancy outcome. This is because whereas some investigators have shown statistically significant association between unemployment and PTD [84–86], others have shown no effect [87–89]. The unemployed pregnant woman is more likely to be socially disadvantaged, having low income, and being unmarried [90]. These exposures can bring about adverse pregnancy outcomes. For example, in our study sample, being unmarried was a key predictor of LBW. The potential contributory factors may include social and economic deprivation and psychological stress, depression and low levels of practical support [91].

As demonstrated in numerous studies including this one, these findings buttress the suggestion that measurement of the content as well as timing and frequency of care of ANC gives a better assessment of the risk of preterm birth than assessment of the number of antenatal visits alone. Therefore, in low- and middle-income countries in particular, health authorities should endeavour to ensure that the provision and utilization of timely and frequent ANC services is sustainable.

### Limitations of the study

As a cross sectional study, associations rather causal relationships were implied in the results. Some information was based on mothers’ recall but some of participants may not exactly remember some past event. This may lead to information bias. However, an effort was made to reduce the recall bias by recruiting postpartum women who had recently delivered in past 12 months of the study. For completeness, the study could have addressed other adverse outcomes such as intrauterine growth restriction and still birth, but we were unable to collect the data for that. The data analysis is thus limited to LBW and preterm birth which are the key adverse pregnancy outcomes that contribute significantly to neonatal morbidity and mortality.

Although several critical confounding covariates were included in the analyses, the observed associations might be influenced by residual confounding because of other unmeasured factors such as performance of intensive physical work during pregnancy and infections such as HIV and malaria. The study findings regarding a minimum of four ANC visits may not be comparable to the current WHO recommended eight ANC visits for an uncomplicated pregnancy [92].

## Conclusion

Total adherence to the recommended ANC initiation, attendance and receipt of essential services had greater protection against PTD and LBW, compared to any single element/component of ANC. Furthermore, adequate ANC utilization had greater protection against PTD than LBW.

## Data Availability

The data associated with the study is available from corresponding author upon reasonable request.
